# Whole genome sequence of a long-legged fly *Condylostylus longicornis* from Hawaiʻi

**DOI:** 10.3389/fgene.2023.1325213

**Published:** 2023-12-11

**Authors:** Bogdan Sieriebriennikov, Megan L. Porter, Jakub Mlejnek, Keith Short, Fleur Lebhardt, Isabel Holguera, Claude Desplan, Michael W. Perry

**Affiliations:** ^1^ New York University, New York, NY, United States; ^2^ NYU Grossman School of Medicine, New York, NY, United States; ^3^ University of Hawaiʻi at Mānoa, Honolulu, HI, United States; ^4^ Independent Researcher, Loves Park, IL, United States; ^5^ University of California San Diego, San Diego, CA, United States

**Keywords:** *Condylostylus longicornis*, Dolichopodidae, long-legged flies, genome, transcriptome, Hawaiʻi

## Introduction

**FIGURE 1:**
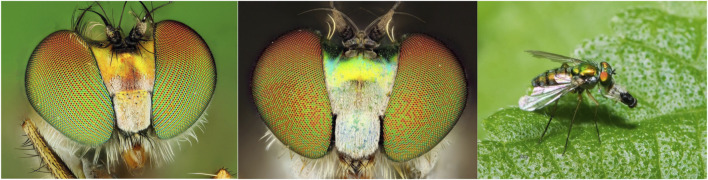
On the left, the head of *C. longicornis* showing alternating columns of two differently colored types of corneal lenses. In the middle, the head of *Chrysosoma* sp. showing a stochastic arrangement of corneal lenses. On the right, a long-legged fly consuming its prey.

Long-legged flies of the family Dolichopodidae represent one of the most species-rich families of the insect order Diptera (Bickel, 2009). The largest number of species is found in the New World tropics (Brown et al., 2018; Pollet et al., 2018; Runyon, 2020; Riccardi et al., 2022), but many species are also present in other regions of the world, and some have been described as tramp species thriving in their non-native ranges (Bickel, 1991; Naglis and Bickel, 2017; Riccardi et al., 2022). Most long-legged flies are predators (Figure 1), and some species are able to hunt common agricultural pests and disease vectors, including mosquitoes (Laing and Welch, 1963), bark beetles (Beaver, 1966) and other insects (Kautz and Gardiner, 2019), making them potential agents of biological pest control. Dolichopodidae were also suggested to be useful as indicators of environmental quality (Pollet, 2010; Gelbič and Olejníček, 2011).

In addition to their ecological significance, long-legged flies have attracted the interest of researchers studying the function and development of sensory organs and the nervous system (Buschbeck and Strausfeld, 1996; Johnston, 2013; Heinloth et al., 2018). Constituent units (ommatidia) of the compound eyes of some dolichopodid species have a highly unusual arrangement (Figure 1): orange-red and green-yellow corneal lenses form alternating vertical rows, and photoreceptor cells that underlie the lenses of different color display different ultrastructure and divergent spectral properties (Trujillo-Cenóz and Bernard, 1972; Stavenga et al., 2017; Ebadi et al., 2018). This ordering has prompted questions about both its functional role, e.g., whether the different rows filter out differently polarized light in high-glare environments, and the underlying developmental mechanisms, considering that most other insects have stochastically arranged eye units in the retina (Johnston, 2013; Wernet et al., 2015; Perry et al., 2016; Heinloth et al., 2018). Finally, Dolichopodidae provide a convenient framework for studying the evolution of sexual behavior as some species display elaborate courtship behavior not seen in related lineages (Zimmer et al., 2003). Thus, long-legged flies are a widespread group of insects whose study has the potential to provide important insight into ecology, pest control, and neurobiology.

Access to high-quality genomic and transcriptomic resources is essential for studying all aspects of biology, from molecular and developmental mechanisms to adaptation to global climate change (McCulloch and Waters, 2023). Despite the abundance and the potential importance of Dolichopodidae, only two genome assemblies are currently available in Genbank for this family: a highly fragmented assembly of *Condylostylus patibulatus* from the Midwestern US (Genbank accession GCA_001014875.1) and a recently released chromosome-level assembly of *Poecilobothrus nobilitatus* from the UK (Genbank accession GCA_947095535.1) created as part of the Darwin Tree of Life Initiative. Here, we report a first high-quality genome assembly and an accompanying transcriptome data set for a species from the tropics - *Condylostylus longicornis* collected in Hawaiʻi. Importantly, the eyes of *C. longicornis* exhibit the unusual arrangement of orange-red and green-yellow corneal lenses, which is only found in a small number of genera (Figure 1). Thus, this species holds particular promise to the studies of eye development and evolution.

## Material collection

**FIGURE 2:**
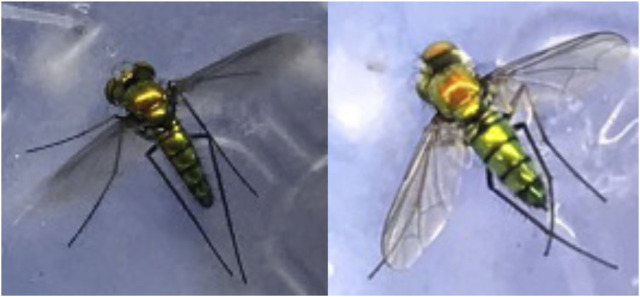
On the left, *C. longicornis* male used for genome sequencing. On the right, *C. longicornis* female used for transcriptome sequencing.

Flies were collected from plant leaves near the Hale Koa Hotel in Honolulu, Hawaiʻi, on 24 November 2020 (Figure 2). *Condylostylus longicornis* was distinguished from other Dolichopodidae that are abundant in the same location, such as *Chrysosoma globiferum*, based on the following characteristics: dark-colored legs, narrow wings canted downward when the fly is in a resting position, rounded abdominal segments, and the dark tip of the abdomen (Naglis and Bickel, 2017). Flies were frozen at −80°C on the day of collection and shipped on dry ice to sequencing facilities.

## DNA sequencing and genome assembly

DNA extraction and genome sequencing was performed at the Center for Advanced Genomics Technology at the Icahn School of Medicine at Mount Sinai. Considering that most species of the suborder Brachycera (to which Dolichopodidae belong) have the XY sex determination system, and males carry different sex chromosomes (Vicoso and Bachtrog, 2015), a male sample was used for the genome sequencing. The whole body of a single individual was pulverized using cryoPREP CP02 (Covaris) and high molecular weight genomic DNA was extracted using MagAttract HMW DNA Kit (Qiagen). The size distribution of the extracted DNA was verified using a Femto Pulse system (Agilent), which showed a single band migrating at 23.6 kb (average size). The DNA was sheared using a g-TUBE (Covaris) at 1,500 g for 2 min 3 times back-and-forth for a total of 6 passes. The average size of the sheared DNA was about 10–11 kb. A HiFi SMRTbell library was prepared using Template Prep Kit 2.0 (PacBio) following the Low DNA Input protocol and sequenced on a Sequel II SMRTcell (PacBio).

Circular consensus sequences (CCS) were generated using PacBio CCS on SMRTLink v10.1 with default parameters. The CCS were assembled using the following strategy. First, mitochondrial reads were identified and assembled using MitoHiFi v3.0.0 (Laslett and Canbäck, 2008; Allio et al., 2020; Uliano-Silva et al., 2023) with the complete mitochondrial genome of *C. luteicoxa* (Genbank accession NC_067856.1) as the reference. Next, the nuclear genome was assembled from the remaining reads using hifiasm v0.16.0 (Cheng et al., 2022) with the --primary flag. Genome completeness of the decontaminated primary assembly was analyzed using BUSCO v5.3.0 (Manni et al., 2021) with a diptera_odb10 database. Contaminant contigs were identified using FCS-GX v0.3.0 (Strope et al., 2023) and removed from the primary assembly. The mitochondrial and the nuclear genomes were merged, and the genome was deposited at NCBI. Adapter sequences identified in the process of the submission were either trimmed off the ends of contigs or removed from the middle of contigs, whereby the contigs containing them were split.

## RNA sequencing and transcriptome assembly

RNA extraction and transcriptome sequencing was performed at New York University. To extract RNA, the head of a single female was separated from the body, and the two body parts were placed in separate tubes containing 0.1 and 2.0 mm BashingBeads (Zymo Research). 1 mL TRIzol (Invitrogen) was added to each tube, and the tissues were homogenized for 2 min at 50 Hz on a TissueLyser LT (Qiagen). The tubes were centrifuged, and the supernatant was mixed with chloroform. Upon phase separation, the aqueous phase was used for RNA cleanup using RNA Clean and Concentrator-5 kit (Zymo Research). The RNA integrity was verified on a TapeStation (Agilent), and four libraries (two from the head RNA and two from the headless body RNA) were prepared using NEBNext Ultra II RNA library prep (New England Biolabs). The libraries were sequenced on NextSeq 500 (Illumina) in MidOutput mode using a 2 × 150 bp run configuration.

The RNA-seq reads were aligned to the RefSeq version of the genome assembly (see below) using STAR v2.7.6a (Dobin et al., 2013). The reads were assembled in the genome-guided mode using Trinity v2.15.1 (Grabherr et al., 2011). Completeness of the transcriptome assembly was analyzed using BUSCO v5.3.0 (Manni et al., 2021) with a diptera_odb10 database. The assembly and the raw data were deposited at NCBI. Assembled transcripts that were either too short or contained adapter or primer contamination were removed during submission.

## Assembly description

The mitochondrial genome assembly had a size of 16,606 bp (greater than 600x coverage) and it contained 37 genes without any frameshifts in the coding sequences (Table 1). Assembling the nuclear genome yielded a 547 Mb (36.3x coverage) primary assembly with 874 contigs, contig N50 of 7.1 Mb, GC content of 26.2% and a 461 Mb alternate assembly with 1,720 contigs, contig N50 of 1.6 Mb, and GC content of 26.7% (Table 1). The size of both the primary and the alternate assemblies is comparable to the size of the publicly available assembly of *C. patibulatus* (452 Mb), although it is considerably smaller than the size of the more distantly related *P. nobilitatus* (944 Mb primary assembly). The GC content is comparable to both *C. patibulatus* and *P. nobilitatus*, which places Dolichopodidae on the lower end of the GC content spectrum among insects (Dennis et al., 2020). Completeness analysis of the primary assembly identified 3,050 (92.8%) BUSCOs, out of which 3,017 (91.8%) were complete and single-copy and 33 (1.0%) were complete and duplicated (Table 1). FCS-GX search identified 17 contaminant contigs in the primary assembly with similarity to the sequences of Gammaproteobacteria. The contaminant contigs were removed from the primary nuclear assembly, which was merged with the mitochondrial assembly and submitted to NCBI. The final assembly (Genbank accession GCA_029603195.2, RefSeq accession GCF_029603195.1) has a size of 544 Mb, it contains 847 contigs with a contig N50 of 7.2 Mb, and the average GC content is 26.5% (Table 1).

**TABLE 1:** Genome and transcriptome assembly statistics.

DNA sequencing: Biological material used	1 male (whole body)
DNA sequencing: technology	PacBio HiFi (Sequel II)
DNA sequencing: number of CCSs	2 M
DNA sequencing: number of bases in CCSs	20 Gb
Mitochondrial genome assembly: size	16,606 bp
Primary nuclear genome assembly: size	547 Mb
Primary nuclear genome assembly: number of contigs	874
Primary nuclear genome assembly: contig N50	7.1 Mb
Primary nuclear genome assembly: GC content	26.2%
Primary nuclear genome assembly: complete BUSCOs	3,050 (92.8%)
Primary nuclear genome assembly: complete and single-copy BUSCOs	3,017 (91.8%)
Primary nuclear genome assembly: complete and duplicated BUSCOs	33 (1.0%)
Primary nuclear genome assembly: fragmented BUSCOs	50 (1.5%)
Primary nuclear genome assembly: missing BUSCOs	185 (5.7%)
Secondary nuclear genome assembly: size	461 Mb
Secondary nuclear genome assembly: number of contigs	1,720
Secondary nuclear genome assembly: contig N50	1.6 Mb
Secondary nuclear genome assembly: GC content	26.7%
Final RefSeq genome assembly: size	544 Mb
Final RefSeq genome assembly: number of contigs	847
Final RefSeq genome assembly: contig N50	7.2 Mb
Final RefSeq genome assembly: GC content	26.5%
Final RefSeq genome assembly: number of genes	14,253
Final RefSeq genome assembly: number of protein-coding genes	12,227
RNA sequencing: biological material used	1 female (head + remaining body)
RNA sequencing: technology	Illumina (NextSeq 500)
RNA sequencing: number of reads	134 M
RNA sequencing: mapping rate to RefSeq genome	55%–61%
Transcriptome assembly: complete BUSCOs	2,425 (73.8%)
Transcriptome assembly: fragmented BUSCOs	89 (2.7%)
Transcriptome assembly: missing BUSCOs	771 (23.5%)

RNA sequencing yielded 134 million reads. Completeness analysis of the transcriptome assembly identified 2,425 (73.8%) BUSCOs (Table 1). The RNA-seq data deposited to NCBI facilitated the creation of a RefSeq gene annotation set (GCF_029603195.1-RS_2023_04) produced using the Eukaryotic Genome Annotation Pipeline (Thibaud-Nissen et al., 2013). The annotation set contains 14,253 genes, which includes 12,227 protein-coding and 1,617 non-coding genes (Table 1).

## Conclusion

In summary, we have created a highly contiguous genome assembly of *C. longicornis*. It is the third genome assembly for the family Dolichopodidae, and the only long-legged fly genome assembly that used biological material collected in the tropics, which harbor the greatest species diversity, yet remain understudied. The genome assembly of *C. longicornis* will serve as a valuable resource for those who study eye development and behavior of long-legged flies. In addition to the nuclear genome, we report the complete mitochondrial genome assembly, which is useful for phylogenetic inferences. Finally, we provide RNA-seq data which facilitate gene structure annotations and are often used in studies on gene evolution.

## Data Availability

The datasets presented in this study can be found in online repositories. The names of the repository/repositories and accession number(s) can be found below: https://www.ncbi.nlm.nih.gov/, PRJNA932838 https://www.ncbi.nlm.nih.gov/, PRJNA932843 https://www.ncbi.nlm.nih.gov/genbank/, JAQSLO000000000 https://www.ncbi.nlm.nih.gov/, GCF_029603195.1.
